# Light and electron microscopy of the pharynx and gastrodermis of the monogenean gill parasite *Macrogyrodactylus clarii* from the catfish *Clarias gariepinus* (Burchell, 1822)

**DOI:** 10.1007/s00436-025-08485-1

**Published:** 2025-04-08

**Authors:** Mohamed Mohamed El-Naggar, Safaa Zaky Arafa, Samir Ahmed El-Abbassy, Jo Cable

**Affiliations:** 1 Zoology Department, Faculty of Science, Mansoura University, Mansoura, Egypt; 2 Department of Biological Sciences, College of Science, King Faisal University, Eastern Province, Al-Ahsa, Saudi Arabia; 3 Zoology Department, Faculty of Science, Damanhour University, Damanhour, Egypt; 4 School of Biosciences and Water Research Institute, Cardiff University, Cardiff, CF10 3AX UK

**Keywords:** Monogenea, *Macrogyrodactylus clarii*, Ultrastructure, Digestive system, *Clarias gariepinus*

## Abstract

The functional morphology of the digestive system in monogeneans is important in understanding feeding behaviour, dietary intake, and metabolic activity of the caecal epithelium. The present study used light and transmission electron microscopy to reveal detailed structure of the pharynx and gastrodermis of the viviparous gill monogenean *Macrogyrodactylus clarii* to compare with the congeneric skin monogenean *Macogyrodactylus congolensis* and other gyrodactylids. The basic components of the pharynx and gastrodermis of *M. clarii* are similar to *M. congolensis*. The pharynx comprises two regions: an anterior highly muscular region and a posterior glandular syncytium with 6 protrusible papillae. The syncytial epithelium lining the mouth and pharyngeal cavity is a modified layer with its own cell bodies, and not an extension of the general body tegument. Eversion of the pharynx has not been observed, but we postulate on the mechanism by which the pharynx and associated muscular structures function during feeding.TEM observations confirmed the similarity between the digestive system of *M. clarii* and *M. congolensis* with notable exceptions: the absence of melanin pigments and microorganism-like structures, presence of unique gastrodermis outgrowths, fibrotic vacuoles and small electron-dense secretory bodies and finally formation of deep intestinal crypts with numerous parallel intestinal lamellae in *M. clarii*. We discuss the possible roles of the luminal lamellar loops, gastrodermis outgrowths, deep intestinal crypts, lipid-like droplets, fibrotic vacuoles and different types of vesicles and vacuoles present in the gastrodermis cytoplasm.

## Introduction

In Egypt and other African countries, the freshwater catfish *Clarias gariepinus* (Burchell, 1822) is known to be parasitized by several monopisthocotylean monogeneans including *Gyrodactylus* Nordmann, 1832, *Macrogyrodactylus* Malmberg 1957, *Quadriacanthus* Paperna, 1961, and *Paraquadriacanthus* Ergens 1988. Two species of *Macrogyrodactylus* were recorded and described from *C. gariepinus* in Egypt, *Macrogyrodactylus clarii* Gusev, 1961 from the gills by El-Naggar and Serag (1987) and *Macrogyrodactylus congolensis* (Prudhoe, 1957) Yamaguti, 1963 from the skin and fins by El-Naggar et al. (1999)*.* Gyrodactylids are unique among monopisthocotylean monogeneans in being viviparous with a direct life cycle that lacks free swimming ciliated larva (oncomiracidium). Each *Macrogyrodactylus* parasite possesses up to four embryos in utero, in various stages that develop by polyembryony (Cable et al. 2002). Gyrodactylids are pathogens of both marine and freshwater fishes (Johnsen et al. 2000; Paladini et al. 2009; El-Naggar et al. 2016). During feeding on the skin or gills of their host fish, gyrodactylids release proteolytic enzymes (Buchmann 1998), which break down the epidermis of the host. Active feeding of gyrodactylids causes lesions in the host skin or gills (Cable et al. 2002; Arafa et al. 2003, 2013; Bakke et al. 2007; Maduenyane et al. 2022a).

Most previous studies on *M. congolensis* and *M. clarii* concentrated on their morphology (El-Naggar et al. 2001a, 2004, 2007, 2019, 2020; Arafa et al. 2003, 2014; El-Naggar and Cable 2007; Arafa 2011; Maduenyane et al. 2022b), ecology (El-Naggar et al. 1999, 2001b), mode of attachment and impact on host tissues (Arafa et al. 2009; El-Naggar et al. 2016; Mahdy et al. 2022; Maduenyane et al. 2022a), and molecular and phylogenetic analyses (Truter et al. 2021). Little attention has been paid to their feeding and gastrodermis ultrastructure (Arafa et al. 2013).

Monopisthocotylean and polyopisthocotylean monogeneans differ in their diet and organization of the gastrodermis although there are exceptions. Monopisthocotyleans typically feed on host epidermal cells and mucus, and their gastrodermis constitutes a single type of digestive cell or syncytium (Halton and Stranock 1976; Arafa et al. 2013), whereas polyopisthocotyleans feed mainly on blood, and the gastrodermis consists of two cell types: digestive cells that alternate with a connecting syncytium (Allen and Tinsley 1989; Poddubnaya et al. 2015; Cable and El-Naggar 2021). However, blood pigments have been detected in the gut of some monopisthocotyleans (Kearn 1963; Fournier 1978; Buchmann et al. 1987), epithelial cells and mucus in some polyopisthocotyleans (du Preez and Verneau 2020) and both blood and epithelial cells in the gut lumen of polyopisthocotylean species (Allen and Tinsley 1989; Cable and El-Naggar 2021).

Although the gastrodermis ultrastructure was studied in many monopisthocotyleans (see for example Cable et al. 1997, 2002; Arafa et al. 2013) and polyopisthocotyleans (Bogitsh 1993; Poddubnaya et al. 2015; Cable and El-Naggar 2021), little attention has been paid to the ultrastructure of the digestive system of *Macrogyrodactylus* spp. To date, there has been only one study on the ultrastructure of the intestine of *M. polypteri* (see Cable et al. 1997) and another on the feeding process and gastrodermis of *M. congolensis* (see Arafa et al. 2013). Therefore, the present study was conducted to investigate the pharynx and gastrodermis ultrastructure of the closely related gill parasite *M. clarii* of *C. gariepinus* in Egypt. This permits a comparison between the digestive system ultrastructure of *Macrogyrodactylus* spp. inhabiting two different microhabitats, the gill parasite *M. clarii* (present study) and skin parasites *M. polypteri* and *M. congolensis* (described previously by Cable et al. 1997; Arafa et al. 2013).

## Materials and methods

Specimens of the catfish *Clarias gariepinus* (Burchell, 1822) were collected from Damietta branch of the Nile River, near Mansoura, Dakahlyia province, Egypt. Fish were transferred to the parasitology laboratory at Zoology Department, Faculty of Sciences, Mansoura University and kept alive in a tank with river water at 25 °C provided with an air pump. Fish (*N* = 20) were euthanized by cranial destruction (inserting the tip of a fine needle into the brain) and the gills were isolated, placed in Petri dishes containing filtered river water and searched for *Macrogyrodactylus clarii* with a dissecting microscope. Living specimens (*N* = 10) were transferred in a drop of water to a clean microscope slide, flattened under a coverslip and examined using a phase-contrast microscope. Other parasites (*N* = 10) were flattened, stained with light green and eosin (see Arafa 1999) and examined with light and phase-contrast microscopes. Semithin, toluidine blue-stained sections for light microscopy and ultrathin sections for transmission electron microscopy (TEM) were prepared as follow: live parasites (*N* = 10) were washed repeatedly in filtered river water, fixed in 2.5% glutaraldehyde buffered in 2.5% sodium cacodylate (0.1 M containing 3% sucrose and 0.1 M CaCl_2_) to pH 7.3 for 2 h before rinsing in several changes of the same cold buffer for 1 h. Specimens were then post-fixed in freshly prepared 1% osmium tetroxide in 0.1 M sodium cacodylate-HCl for 30 min at 4 °C, stored in the buffer overnight and then dehydrated in an ascending series of ethanol followed by propylene oxide. Dehydrated specimens were embedded overnight at 60 °C in capsules containing Spurr resin. Semithin sections through the pharynx, oesophagus and gastrodermis were cut with an LKB ultramicrotome using glass knives at a thickness of 1 μm, stained with 1% toluidine blue in 1% borax, mounted in DPX and examined using bright field microscopy with oil immersion. For TEM, ultra-thin sections were cut through the pharynx and different regions of the digestive system at 70–90 nm using LKB ultramicrotome and diamond knife. The sections were mounted on single hole coated copper grids and stained in 1–2% uranyl acetate for about 30 min followed by 2–3% lead citrate for about 5 min. Stained sections were examined using a JEOL 100SX transmission electron microscope operating at 80 kV, in the imaging hub of the School of Biosciences, Cardiff University.

## Results

### Light microscopy

**Fig. 1:**
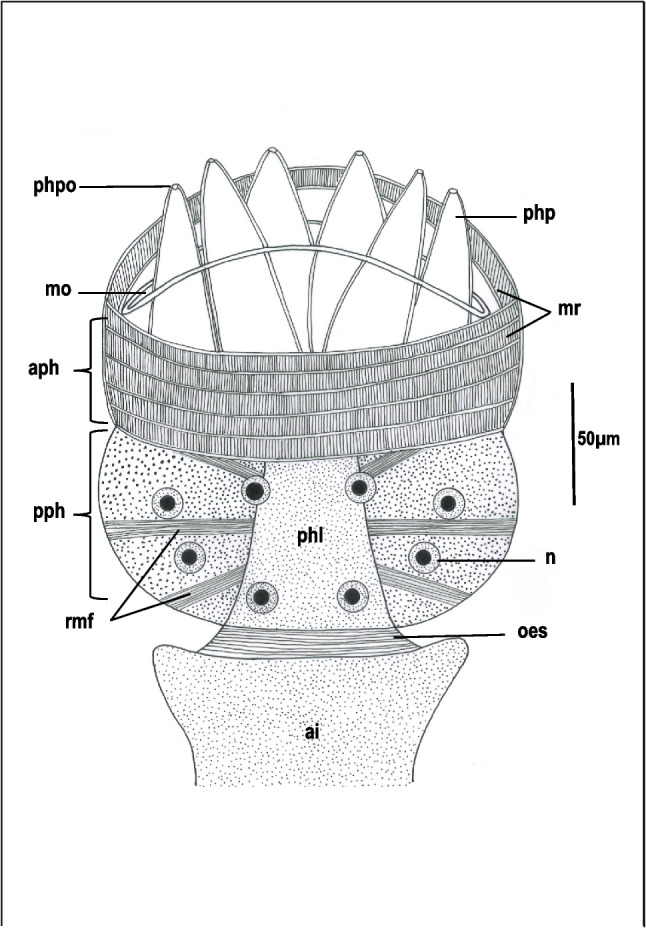
Diagram showing well-flattened pharynx, oesophagus (oes) and anterior region of the intestine (ai) of *Macrogyrodactylus clarii*. aph, Anterior region of the pharynx; mo, mouth; mr, muscle rings; n, nucleus; pph, posterior region of the pharynx; phl, pharyngeal lumen; php, pharyngeal papilla; phpo, pharyngeal papilla opening; rmf, radial muscle fibers

**Fig. 2:**
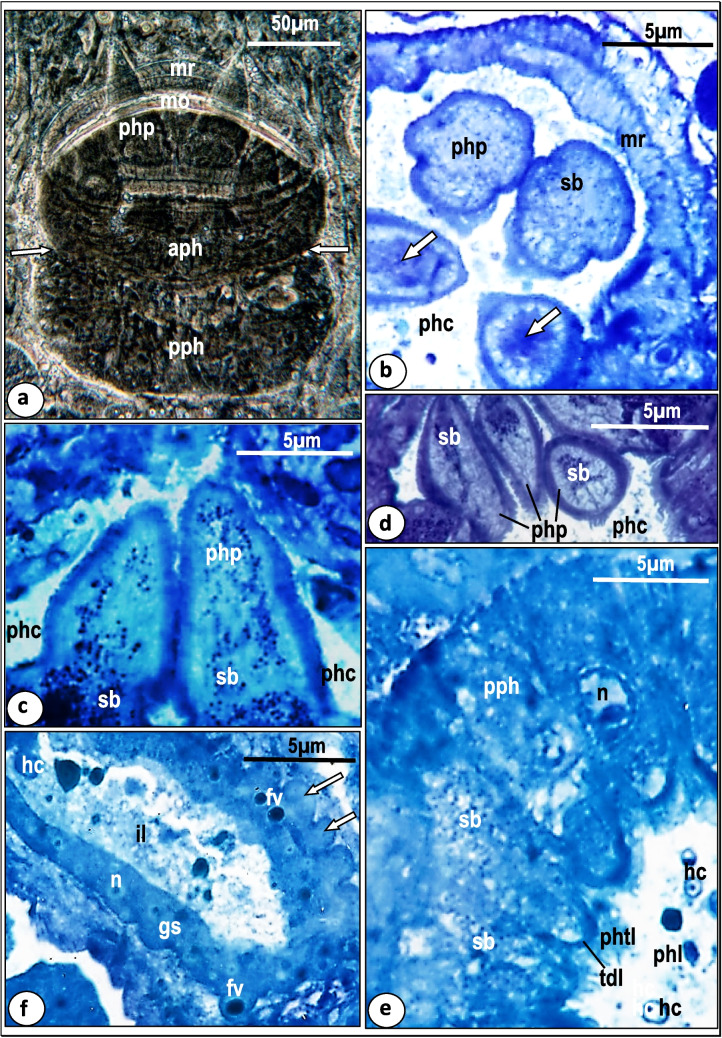
Phase-contrast micrograph of a well flattened pharynx (**a**) and light micrographs of toluidine blue-stained sections through alimentary tract (**b-e**) of *Macrogyrodactylus clarii.* **a** Pharynx showing that its anterior region (aph) with muscle rings (mr) and its posterior region (pph) are demarcated from each other by a superficial constriction (arrows) and some pharyngeal papillae (php) project from mouth (mo). **b** Anterior region of pharynx showing pharyngeal papillae (php), rings of muscle fibers (mr), pharyngeal cavity (phc), spherical secretory bodies (sb) and spacious sac on terminal region of pharyngeal papilla (arrow).**c** Longitudinal section through the pharyngeal papillae (php) showing secretory bodies (sb) arranged in nearly parallel lines. phc, Pharyngeal cavity. **d** Terminal regions of pharyngeal papillae (php) packed with secretory bodies (sb). phc, Pharyngeal cavity. **e** Posterior region of the pharynx (pph) showing that it is a glandular syncytium containing spherical secretory bodies (sb) and nuclei (n) and lined with pharyngeal tegumental layer (phtl) underlined by a thick dense layer (tdl). hc, Host cell; phl, pharyngeal lumen. **f** The gastrodermis (gs) with nuclei (n) and food vacuoles (fv) and its outer surface forms a zigzag-like structure (arrows) while its lumen (il) contains some host cells (hc)

In flattened specimens, the alimentary tract of *Macrogyrodactylus clarii* consists of a crescent-shaped mouth, large pharynx, short oesophagus, and an intestine differentiated into an anterior median, wide region which bifurcates into two long intestinal diverticula running posteriorly a short distance from the anterior limit of the haptor. The pharynx comprises an anterior highly muscular region and a posterior mainly glandular region demarcated from each other by a superficial constriction (Figs. 1 and 2a). The wall of the anterior region of the pharynx, which encloses the pharyngeal cavity, is made up of 5–6 muscle rings separated from each other by circular muscle fibers (Figs. 1 and 2a, b). Each ring consists of parallel, radially arranged muscle fibers (Figs. 1 and 2b). The posterior region of the pharynx forms a glandular syncytium containing eight large nuclei and spherical secretory bodies (Figs. 1 and 2e). In toluidine blue-stained sections, the lumen of the inner surface of the posterior pharynx exhibits a zigzag-like structure. This is covered by the pharyngeal lining layer, which is supported by a thick, dense underlying layer (Fig. 2e). Six conical-shaped, muscular papillae extend as cytoplasmic processes from the anterior extremity of the glandular syncytium into the pharyngeal cavity where their distal regions, in most flattened specimens, protrude through the mouth opening (Figs. 1 and 2a, b). Each papilla contains spherical secretory bodies that appear in nearly parallel lines at its distal region and open into the exterior through a spacious sac lying on the apex of the papilla (Figs. 1 and 2c, d). The gastrodermis lining the intestinal lumen forms a single syncytial layer containing numerous nuclei and food vacuoles (Fig. 2f). In some regions of the intestine, the basal portion of the gastrodermis is zigzag-like in profile while its lumen contains some host cells (Fig. 2f).

### Transmission electron microscopy (TEM)

**Fig. 3:**
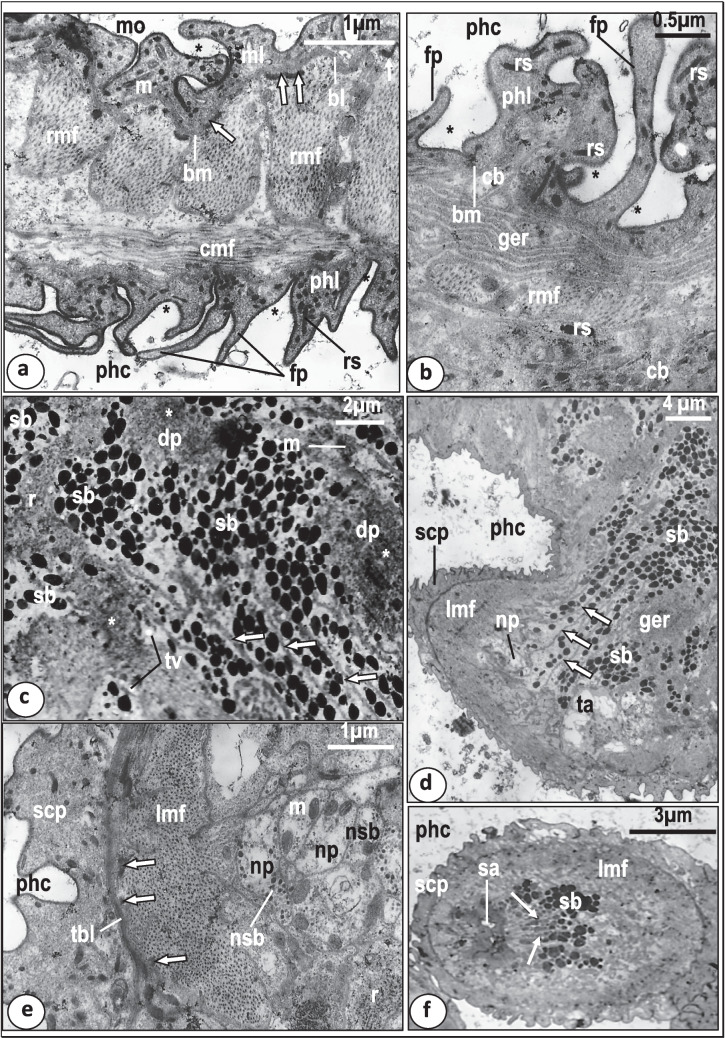
Transmission electron micrographs (TEM) through the anterior region of the pharynx of *Macrogyrodactylus clarii*. **a** The wall of the anterior region of the pharynx showing circular muscle fibers (cmf), radial muscle fibers (rmf), which form electron-dense thickenings (arrows) at the basal lamina (bl) of the mouth’s syncytial lining (ml). Note that the mouth’s (mo) syncytial lining (ml) and the pharyngeal syncytial layer (phl) lining the pharyngeal cavity (phc) form deep infoldings (*) and finger-like processes (fp) which are more numerous in the pharyngeal lining layer. bm, basal plasma membrane; m, mitochondrion; rs, rod-shaped bodies. **b** Magnified part of the syncytial layer (phl) lining the pharyngeal cavity (phc) with numerous finger-like processes (fp) that form deep folds (*). Note the presence of a part of the cell body (cb) that provides this layer with rod-shaped secretion (rs). bm, Basal membrane; ger, granular endoplasmic reticulum; rmf, radial muscle fibers. **c** The glandular syncytial cytoplasm with spherical, highly electron-dense secretory bodies (sb) and membranous partitions (arrows). Note that the highly electron-dense regions (white star) are packed with fine dense particles (dp). m, Mitochondrion; r, ribosomes; tv, small translucent vesicles. **d** The basal region of the pharyngeal papilla with secretory bodies (sb) arranged in many rows that are separated from each other by membranous partitions (arrows). Note the presence of a nerve process (np) associated with the longitudinal muscle fibers (lmf) lying underneath the syncytial covering of the pharyngeal papilla (scp). ger, Granular endoplasmic reticulum; phc, pharyngeal cavity; ta, translucent area. **e**.Magnified part of the basal region of the pharyngeal papilla showing its syncytial covering (scp) underlined by a thick basal lamina (tbl) and nerve processes (np) containing small neurosecretory bodies (nsb) associated with the longitudinal muscle fibers (lmf). Note the presence of electron-dense thickenings (arrows) between the thick basal lamina and longitudinal muscle fibers. m, Mitochondrion; phc, pharyngeal cavity; r, ribosomes. **f** The apex of the pharyngeal papilla with a terminal sac-like structure (sa) and secretory bodies (sb) arranged in nearly radial rows (arrows) towards the terminal sac. lmf, Longitudinal muscle fibers; phc, pharyngeal cavity; scp, syncytial cytoplasmic layer covering the pharyngeal papilla

The crescent-shaped mouth leads directly into the pharyngeal cavity of the anterior muscular region of the pharynx (Fig. 1). The inner surface of both the mouth opening and the pharyngeal cavity is covered with a syncytial lining layer that rests on a plasma membrane associated with a thin basal lamina (Fig. 3a, b). This layer is apparently folded and forms many finger-like processes enclosing infoldings of the outer surface membrane that extend deeply into the syncytial layer as far as the basement membrane (Fig. 3a, b). However, these finger-like processes are more numerous in the pharyngeal cavity than in the mouth opening (Fig. 3a, b). The outer surface membrane of this layer is covered with a conspicuous, thick, electron-dense layer (Fig. 3a, b). The cytoplasm of the syncytial layer is fibrous and moderately electron-dense and contains rod-shaped, highly electron-dense secretory bodies and few elongated mitochondria (Fig. 3a, b). There is no evidence indicating the presence of Golgi bodies, endoplasmic reticulum or ribosomes in the mouth or pharyngeal lining layers. Dispersed portions of the cell bodies producing the rod-shaped bodies are seen in close contact with the lining syncytial layer (Fig. 3b). Each ring of the muscular anterior region of the pharynx consists of a continuous layer of circular muscle fibers and blocks of radial muscle fibers occupying most of the pharyngeal wall (Fig. 3a). Small electron-dense thickenings (sarcodesmosomes) are found between the periphery of the radial muscle blocks and basal lamina (Fig. 3a).

The glandular region of the pharynx forms a syncytial cytoplasm, mostly moderately electron-dense interspersed by sparse radial muscle fibers (Fig. 3d), but with some highly electron-dense cytoplasmic regions packed with fine dense particles and a few translucent regions that lack secretory inclusions (Fig. 3c, d). Some dispersed radial muscle fibers were observed in the peripheral regions of the glandular syncytium (Fig. 3d). The moderately electron-dense regions are packed with spherical, highly electron-dense, secretory bodies (Fig. 3c, d). Also, a few translucent vesicles and mitochondria are present in these areas (Fig. 3c, d). The spherical secretory bodies, particularly in the cytoplasmic regions lying close to the basal portions of the pharyngeal papilla, are arranged in many rows which are separated by membranous partitions (Fig. 3c, d). The basal region of each pharyngeal papilla is supported by longitudinal muscle bands which are often innervated by nerve processes containing small neurosecretory bodies, ribosomes, and mitochondria (Fig. 3d, e). The syncytial covering of the pharyngeal papilla at its base resembles, in structure, the lining of the pharyngeal cavity with some modifications: fewer finger-like processes and infoldings, and the basal lamina is thicker and highly, electron-dense (Fig. 3e). In the distal region of each pharyngeal papilla, the secretory bodies are arranged in near parallel rows which run anteriorly in a radial direction towards the apex of the papilla where they open into a single sac ensheathed by a thin layer of high electron-density (Fig. 3f). The sac opens to the exterior through a single opening.

**Fig. 4:**
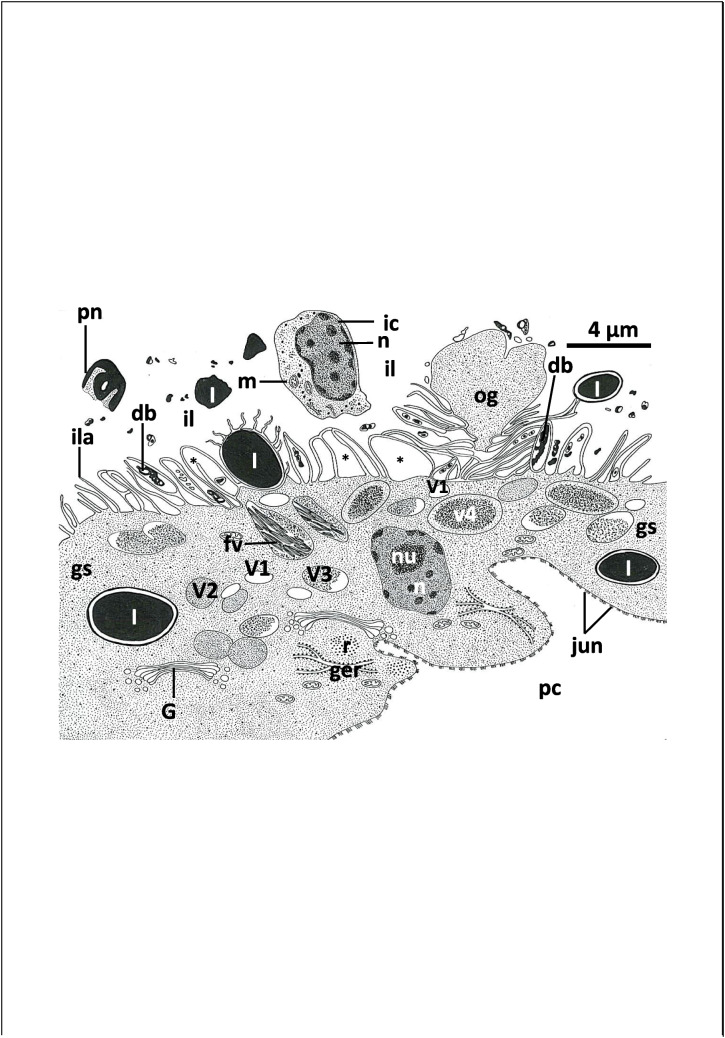
Diagram showing the syncytial gastrodermis (gs) and intestinal lumen (il) ultrastructure of *Macrogyrodactylus clarii*. db, Electron-dense bodies; fv, fibrotic vacuole; Go, Golgi bodies; ger, granular endoplasmic reticulum; ic, intact host cell; ila, intestinal lamellae; jun, junctions between the gastrodermis membrane and that of the neighboring parenchymal cell (pc); l, lipid-like droplet; m, mitochondrion; n, nucleus; nu, nucleolus; og, outgrowth of gastrodermis; pn, pyknotic host-cell nucleus; r, ribosomes; V1, V2, V3 and V4 vesicles/vacuoles in the gastrodermis

**Fig. 5:**
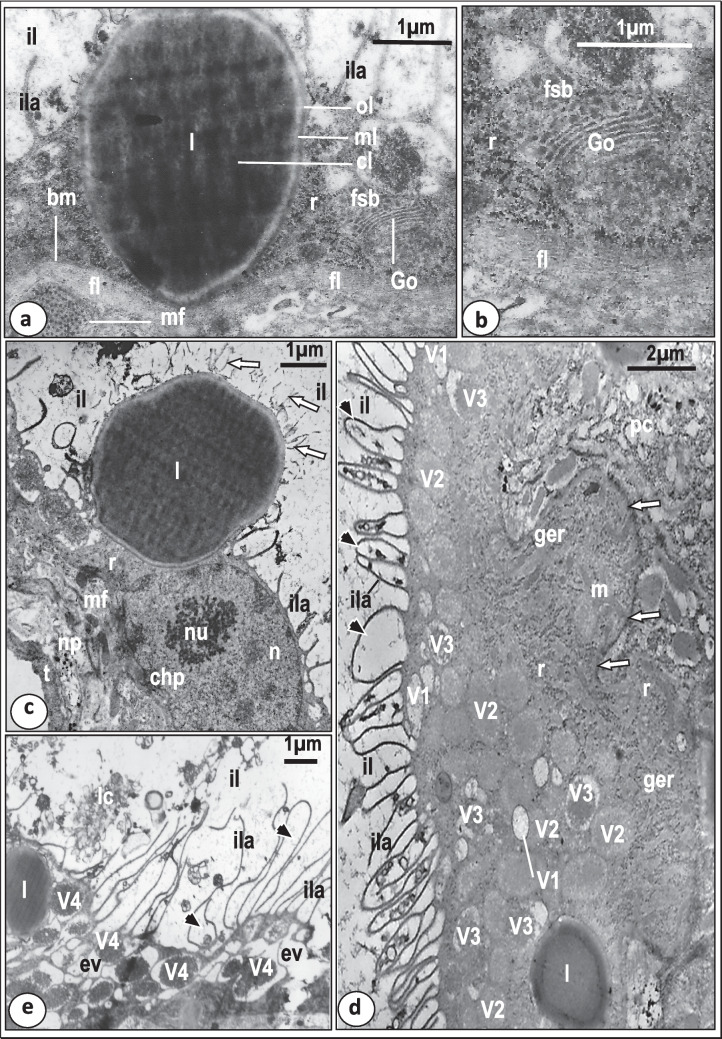
Transmission electron micrographs (TEM) showing the gastrodermis of *Macrogyrodactylus clarii*. **a** Part of the gastrodermis containing large lipid-like droplet (l) consisting of 3 layers, an outer thin, highly electron-dense layer (ol), a median layer with low electron density (ml) and a central mass of highly electron-dense matrix (cl).The gastrodermis cytoplasm contains Golgi bodies (Go), ribosomes (r) and fine secretory bodies (fsb) and its luminal surface forms intestinal lamella (ila). fl, Fibrous layer; il, intestinal lumen; mf, muscle fibers; bm, basement membrane. **b** Magnified part of the gastrodermis showing Golgi bodies (Go) with 4–6 narrow cisternae, neighboring fine secretory bodies (fsb) and ribosomes (r). fl, Thick fibrous layer underneath the gastrodermis. **c.**Thin region of the gastrodermis containing oval-shaped nucleus (n) with nucleolus (nu) and chromatin patches (chp), nerve process(np) associated with muscle fibers (mf), ribosomes(r), and large lipid-like droplet (l) covered by degenerated lamellae (arrows). il, Intestinal lumen; ila, intestinal lamella; t, general body tegument. **d** Thick region of gastrodermis containing endocytotic translucent vesicles (V1), abundant vacuoles with homogenous, moderately electron dense substance (V2), vacuoles filled with homogeneous, highly electron-dense substance with a peripheral zone of low electron density (V3), granular endoplasmic reticulum (ger), mitochondrion(m), ribosomes (r) and lipid-like droplets (l). Note that some intestinal lamellae (ila) recurve and rejoin the luminal surface to form loop-like structures (black arrow heads). Note highly electron-dense junctions (arrows) between the gastrodermis membrane and that of the neighboring parenchymal cell (pc). il, Intestinal lumen. **e.** Luminal surface of the gastrodermis with many long intestinal lamellae (ila), some of which form loop-like structures (black arrow heads) projecting into the intestinal lumen (il). Note that the gastrodermal cytoplasm contains V4 vacuoles filled with highly-electron-dense matrix, empty vacuoles (ev), lipid-like droplets (l). lc, Homogeneous luminal content

**Fig. 6:**
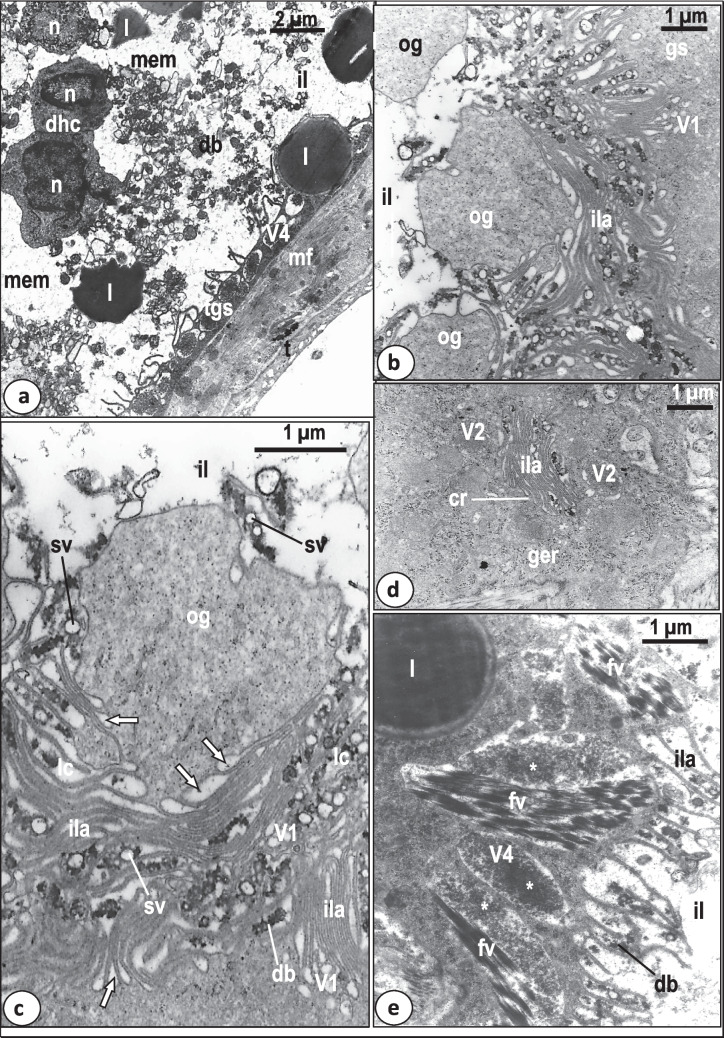
Transmission electron micrographs (TEM) showing gastrodermis and intestinal luminal contents of *Macrogyrodactylus clarii*. **a** Thin gastrodermis (tgs) packed with highly electron-dense cytoplasm, only one row of V4 vacuoles and lipid-like droplet (l). Note that the intestinal lumen (il) contains lipid-like droplets (l), partially digested host tissue cells (dhc) with intact nuclei (n), highly-electron-dense bodies (db) and homogeneous moderately electron-dense material (mem). mf, Muscle fibers; t, general body tegument. **b** Luminal surface of the gastrodermis with numerous parallel and interdigitated long intestinal lamellae (ila) and characteristic cytoplasmic outgrowths (og) protruding into the intestinal lumen (il). gs, Gastrodermis; V1, endocytotic, translucent vesicles. **c** Magnified part of the gastrodermis showing outgrowth (og), numerous parallel and interdigitating long intestinal lamellae (ila), and luminal contents (lc) with small vesicles (sv) and electron-dense bodies (db). Note the apical plasma membrane invaginates into the gastrodermis cytoplasm to form translucent endocytotic vesicles (arrows). il, Intestinal lumen; V1, vesicles with translucent content. **d** Gastrodermis cytoplasm with a deep intestinal crypt (cr) containing numerous parallel intestinal lamellae (ila). ger, Granular endoplasmic reticulum; V2, vacuole with homogenous, moderately electron dense substance. **e** Gastrodermis containing lipid-like droplet (l), V4 vacuoles with electron-dense contents (white star), and fibrotic vacuoles (fv) filled with bundles of highly electron-dense presumed fibrotic structures. Note that some of (fv) vacuoles contain electron-dense contents (white star) similar to those found in the V4 vacuoles. db, Electron-dense bodies; il, intestinal lumen; ila, intestinal lamellae

The intestinal wall consists of a single layer, syncytial gastrodermis resting on a plasma membrane, underlined by a relatively thick fibrous layer (Figs. 4 and 5a). This layer is underlined and sometimes penetrated by dispersed muscle fibers (Figs. 4 and 5a). The thickness of the gastrodermis varies along the length of the caecum from 3–9 μm (Figs. 5d and 6a). The cytoplasm of the syncytial gastrodermis contains roughly spherical nuclei, each possessing a conspicuous nucleolus with a highly electron-dense matrix and chromatin patches under the nuclear membrane (Figs. 4 and 5c). Few mitochondria, granular endoplasmic reticulum with conspicuous cisternae and clusters of ribosomes are also present (Fig. 5a, b, c, d). Golgi bodies are clearly visible and distributed all over the cytoplasm, each comprising 4–6 parallel cisternae terminating with dilated vesicles (Fig. 5a, b). Fine secretory bodies are evident in close contact with Golgi bodies (Fig. 4, 5a, b). In the thick gastrodermis, some basal regions expand into the neighboring parenchymal tissues and highly electron-dense junctions are formed between the gastrodermis membrane and the parenchymal cell membrane (Figs. 4 and 5d). The luminal surface of the gastrodermis is covered by an outer plasma membrane which protrudes into the intestinal lumen forming many, mostly parallel lamellae (Figs. 4 and 5a, c, d, e). These lamellae are mostly unbranched and vary in length (1–5 μm) and number along the length of the gastrodermis (Figs. 4 and 5a, c, d, e). Many lamellae recurve and rejoin the luminal surface of the gastrodermis forming loop-like structures enclosing material similar to that found in the intestinal lumen (Fig. 5d, e).

Large densely stained inclusions that appear similar to lipid droplets (diameter 1–4 μm), were found in the gastrodermis (Fig. 5a, c, d, e) and intestinal lumen (Fig. 6a). Most had a roughly circular profile, the remainder with an irregular shape (Figs. 5a, c and 6a). In section, each lipid-like droplet consists of 3 layers, an outer thin, highly electron-dense layer, followed by a considerably thicker median layer with low electron density and a central mass of highly electron-dense matrix constituting the main bulk of the droplet (Fig. 5a, c). In many sections, these presumed lipid droplets are located superficially, very close to the luminal surface and sometimes found in projections of the gastrodermis with intestinal lamellae and thin layer of gastrodermis cytoplasm facing the intestinal lumen (Fig. 5a, c). Some of the intestinal lamellae present on the surface of these projections containing presumed lipid projections appeared to be in a degenerated condition (Fig. 5c). Other lipid-like particles occurred deep in the gastrodermis cytoplasm (Fig. 5d). Thus, these presumed lipid droplets are not only found in the gastrodermis and intestinal lumen but also in parenchymal and haptor tissues.

In regions of the intestine that are in close contact with the uterus, the gastrodermis appears thinner (1–1.5 μm), its cytoplasm is highly electron-dense and the intestinal lamellae are shorter (Fig. 6a). In some sections of the intestine, the apical part of the gastrodermis bears outgrowths that protrude into the intestinal lumen (Fig. 6b, c) and contains similar components to the gastrodermis cytoplasm but lacking Golgi bodies, endoplasmic reticulum or mitochondria (Fig. 6c). In other sections, the outer surface of most of these cytoplasmic outgrowths appear to have no luminal lamellae while their inner surfaces form a great number of intestinal lamellae, some of which form a network of loops and interdigitate to enclose a great part of the luminal contents while others run in a parallel direction (Fig. 6b, c). In other sections of the gastrodermis, deeper crypts which are provided with intestinal lamellae were observed surrounded by gastrodermis cytoplasm (Fig. 6d).

**Fig. 7:**
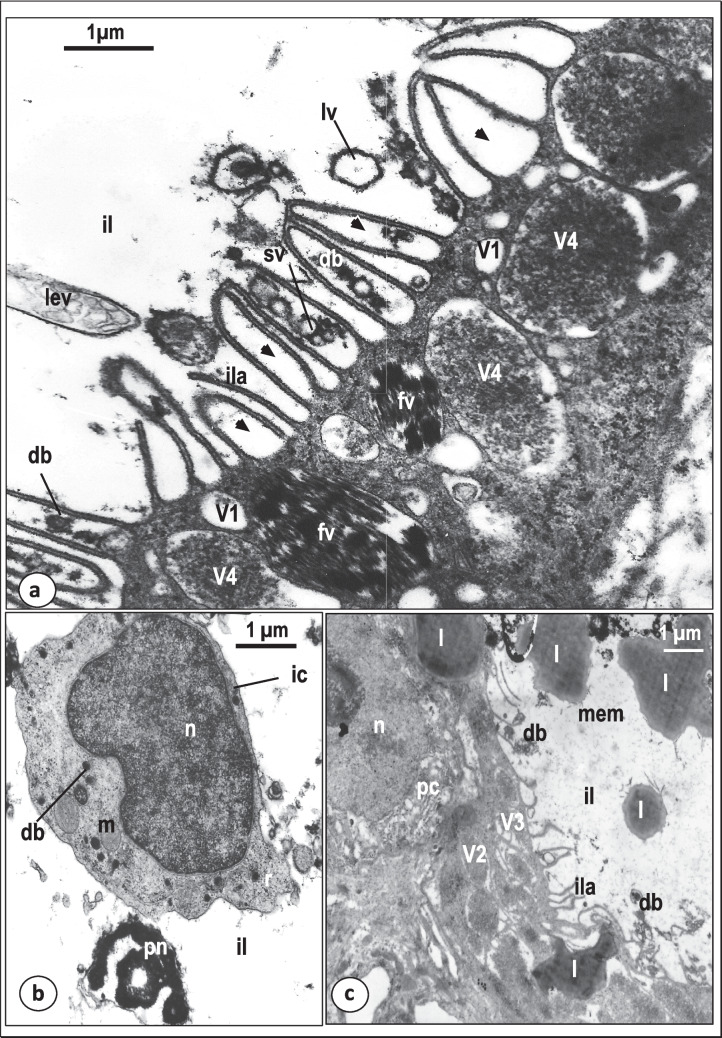
Transmission electron micrographs (TEM) showing gastrodermis and intestinal luminal contents of *Macrogyrodactylus clarii*. **a** Magnified part of the thin gastrodermis showing V1 vesicles, V4 vacuoles, fibrotic vacuoles (fv) and intestinal lamellae (ila) with loop-like structures (black arrow heads). Note that the intestinal lumen (il) contains electron-dense bodies (db), small rounded translucent vacuoles (sv), large rounded translucent vacuoles (lv) and large elongated vacuoles with many vesicles (lev). **b** Intestinal lumen (il) containing conspicuous intact host cell (ic) with large nucleus (n) and cytoplasm with mitochondria (m), ribosomes (r) and spherical highly electron-dense bodies (db). Note the presence of part of pyknotic host-cell nucleus (pn). **c**.Gastrodermis with irregularly shaped lipid-like droplets (l) in its cytoplasm and in the intestinal lumen (il), V2 and V3 vacuoles, and short intestinal lamellae (ila). Note the presence of highly electron-dense bodies (db) and homogenous moderately electron-dense material (mem) in the intestinal lumen. n, Nucleus of parenchymal cell (pc)

At the luminal surface of the gastrodermis and their outgrowths, the apical plasma membrane invaginates into the cytoplasm forming small endocytotic vesicles (V1), particularly in the regions lying in-between the basal parts of the intestinal lamellae (Fig. 6c). These V1 vesicles are small in size (0.5–1 μm diameter) and appear to separate from the luminal surface into the gastrodermis cytoplasm where they become spherical or oval in shape and contain translucent or moderately electron-dense contents (Figs. 5d and 6b, c). In addition to the V1 vesicles, the gastrodermis cytoplasm contains three other kinds of vacuoles (V2, V3, and V4) (Figs. 5d, e and 6a, c, d, e). The V2 vacuoles are oval-shaped and larger (1–2 µm diameter) than the V1 vesicles. They are filled with homogenous, moderately electron-dense substance (Fig. 5d). In some sections, V1 vesicles are fused with the V2 vacuoles (Fig. 5d). The V3 vacuoles resemble the V2 vacuoles in shape and size but are partially filled with homogeneous, highly electron-dense substance with a peripheral zone of low electron-density (Fig. 5d). In many sections, the V1 vesicles and the V2 and V3 vacuoles are detected close to the luminal surface and in the middle region of the gastrodermis (Fig. 5d). However, none of these vesicles and vacuoles are found in the proximal region of the gastrodermis where the cytoplasm is packed with granular endoplasmic reticulum, mitochondria, ribosomes and Golgi bodies (Fig. 5d). The V4 vacuoles are oval-shaped or spherical in outline and generally larger (1.5–2.5 µm diameter) than V1-3 structures. They contain highly electron-dense, heterogeneous particles embedded in an electron-lucent substance (Figs. 5e and 6e). In many V4 vacuoles, the electron-dense particles form a central mass surrounded by an electron-lucent zone (Figs. 6e and 7a). Some empty vacuoles were seen in the luminal surface beneath the luminal plasma membrane (Fig. 5e). Regions of the gastrodermis cytoplasm containing numerous V4 vacuoles, have no V2 or V3 vacuoles (Fig. 5e). Also, none of the V4 vacuoles are found in the cytoplasmic regions of the gastrodermis packed with V2 and V3 vacuoles (Fig. 5d).

A remarkable feature of the intestinal caecum of *M. clarii* is the presence of large vacuoles containing bundles of highly electron-dense, rod-shaped, presumed fibrotic structures in the gastrodermis cytoplasm (Figs. 6e and 7a). These characteristic vacuoles are not found in the intestinal lumen. They vary in size and shape but mostly are concentrated close to the luminal surface (Figs. 6e and 7a). Some vacuoles are smaller in size (average diameter 1 µm), spherical in outline and contain a single or few bundles of fibrotic structures (Fig. 7a), while others are larger (average length 4 µm), oval-shaped or irregular in outline and contain a large number of bundles (at least 20 in section) of fibrotic structures. These bundles appear to be connected to each other by fine filaments (Fig. 6e). In some sections, electron-dense contents similar to those found in the V4 vacuoles are present in close contact with the fibrotic bundles inside the fibrotic vacuoles (Fig. 6e).

The intestinal lumen contains different contents which include lipid droplets (Figs. 6a and 7c), homogeneous moderately electron-dense material (Fig. 6a), intact and partially digested host tissue cells (Figs. 6a and 7b), highly-electron-dense bodies (Figs. 6a and 7a), small and large electron-lucent vacuoles with an outer electron-dense boundary (Fig. 7a), remains of pyknotic host nuclei (Fig. 7b), large elongated vacuoles packed with small irregularly-shaped vesicles (Fig. 7a). The intact cells in the intestinal lumen have large nuclei, mitochondria and ribosomes (Fig. 7b). No melanin or hematin pigments were detected among the partially digested tissue found in the intestinal lumen or inside the vacuoles of the gastrodermis.

## Discussion

The present study documents the ultrastructure of the digestive system, particularly the pharynx and gastrodermis, of the gill monogenean parasite *Macrogyrodactylus clarii*. Phase-contrast microscopy and toluidine blue-stained sections confirm similarity in the general structure of the digestive system of *M. clarii* with that of *M. congolensis* (see El-Naggar and Serag 1987; Arafa et al. 2013), being composed of a mouth, pharynx, short oesophagus and two intestinal branches. Like *M. congolensis* and other gyrodactylids (El-Naggar et al. 1999; El-Naggar and El-Abbassy 2003; Arafa et al. 2013), the pharynx of *M. clarii* is composed of an anterior muscular region and a posterior mainly glandular syncytium with six protrusible papillae. The glandular syncytium cytoplasm of *M. clarii* differs from that of *M. congolensis* in its differentiation into three distinct areas: moderately electron-dense areas packed with spherical secretory bodies, highly electron-dense areas with small, highly electron-dense particles, and a few translucent areas lacking any kind of secretion, which may have evacuated their secretory bodies.

The pharyngeal papillae of *M. clarii* are equipped with longitudinal muscle fibers and membranous structures which may serve in orientating the spherical secretory bodies towards the wide sac found at the apex of the lamellae and aid in the release of these secretory bodies onto the gill tissues during feeding process. The mechanism of action of the pharynx in *M. clarii* was not studied in living specimens but considering the types and orientation of the muscle fibers observed in the present study and those recorded by El-Naggar et al. (2004), the mechanism can be postulated as follows. Contraction of the radial muscles (rmf) in the posterior pharyngeal region may serve in pushing the secretory bodies towards the pharyngeal papillae, while contraction of the circular muscles (cmf) of the anterior region of the pharynx leads to its elongation. Pharyngeal papillae probably protrude through the mouth opening during relaxation of their longitudinal muscle fibres (lmf) during feeding. Secretory bodies released from the papillae sacs are likely used for extracorporeal digestion of gill tissues and the partially digested host tissues can be withdrawn into the pharyngeal cavity by suction force created by periodic contraction and relaxation of the radial and circular muscles of the anterior muscular region of the pharynx. In *E. soleae*, the pharynx acts as a peristaltic pump by forcing the sea water into the branched intestine (Kearn et al. 1996). In the present work, and for *M. congolensis* (see Arafa et al. 2013), no evidence was found to confirm the eversion of the whole pharynx from the mouth opening, despite this process occurring in other monogeneans (e.g. *Entobdella soleae*, see Kearn 1963; *Cichlidogyrus halli*, see El-Naggar and Khidre 1985; *Gyrodactylus* spp. Mo 1994; Buchmann and Bresciani 1998). In *Gyrodactylus* spp., the pharynx releases digestive proteolytic enzymes (proteases and lysozyme), which are used to break down the fish host skin epidermis (Buchmann and Bresciani 1998).

In *M. clarii*, the mouth opening and pharyngeal cavity are covered with a syncytial cytoplasmic layer containing rod-shaped secretory bodies and provided with finger-like processes which are more numerous in the pharyngeal cavity. This layer resembles the pharyngeal cavity lining of *M. congolensis* except for the finger-like processes (Arafa et al. 2013). The outer membrane of the pharyngeal cavity syncytial lining in *M. congolensis* is highly folded (Arafa et al. 2013, Fig. 2) as in the present study. In comparison with the ultrastructure of the general body tegument (unpublished data by M. M. El-Naggar), it is evident that the syncytial lining of the mouth and pharyngeal cavity of *M. clarii* is a modified syncytial cytoplasmic epithelial layer with its own cell bodies, and not an extension of the general body tegument since the latter contains more numerous and characteristic oval-shaped translucent vesicles in addition to the rod-shaped bodies. Moreover, the body tegument of *M. clarii* has many microvilli (unpublished data by M. M. El-Naggar), a feature not observed in the syncytial lining of the mouth opening and pharyngeal cavity. Bogitsh (1993) reported that the lining of the foregut (mouth, buccal cavity, pharynx and oesophagus) in monogeneans is an extension of the general body covering. This generalized opinion, however, does not apply to *M. clarii* nor to other *Gyrodactylus* species (e.g. Kritsky et al. 1994). In *M. clarii*, the modified syncytial lining of the mouth opening, with its finger-like processes, might serve to increase the surface area available for attachment to the host gill tissue during feeding, while the numerous finger-like processes of the pharyngeal cavity may serve in pushing the partially digested host cells backwards into the pharyngeal lumen.

The luminal surface of the syncytial gastrodermis of *M. clarii* has many mostly unbranched, parallel lamellae which, in many sections, recurve and join the luminal surface to form loop-like structures resembling those found in the gastrodermis of *M. congolensis* (see Arafa et al. 2013). Few and widely spaced, occasionally branched lamellae were reported in the gastrodermis of *Gyrodactylus eucaliae* Ikezaki & Hoffman, 1957 and *Gyrodactylus* sp., but without rejoining the luminal surface and were suggested to increase the surface area available for absorption (Kritsky et al. 1994). Lamellate loops in the intestinal lumen of *M. clarii* can trap and withdraw partially digested material. These loops differ from the balloon-like structures observed in *D. sagittate*, which are larger and contain various luminal contents (Cable and El-Naggar 2021). The loops in *M. clarii* are extensions of the luminal intestinal lamellae, not cytoplasmic in nature as in *D. sagittata,* and might serve in accumulating luminal contents closer to the surface lamellae for absorption (Cable and El-Naggar 2021).

A unique feature of the gastrodermis of *M. clarii* is the apical outgrowths protruding into the intestinal lumen, also found in some polyopisthocotyleans. Poddubnaya et al. (2015) suggested that these structures may be involved in holocrine or apocrine process for eliminating accumulated haematin from the digestive cells. In the present study, there was no evidence to indicate complete separation of the gastrodermis outgrowths so, holocrine or apocrine processes cannot be applied to the gastrodermis of *M. clarii*. These outgrowths of *M. clarii*, with their numerous parallel and interdigitating lamellae and endocytotic vesicles, may increase the luminal surface available for capturing the partially digested mucous and host cells in the lumen.

As in *M. congolensis* (see Arafa et al. 2013), inclusions that appear similar to lipid droplets were observed in the gastrodermis cytoplasm, intestinal lumen, parenchyma and haptor of *M. clarii*. There is no evidence in the present study nor in *M. congolensis* that the endocytotic vesicles (V1) can engulf such large droplets or that they are broken down in the intestinal lumen into small ones; their origin and possible functions are unknown. Grano-Maldonado (2018), however, suggested that part of the lipid material in *G. gasterostei* is derived from maternal reserves, with lipid reserves passed from the mother to the embryo increasing in line with the developmental state of the daughter in utero. In *M. congolensis*, Arafa et al. (2013) suggested a host tissue origin, but with no evidence of this.

Four kinds of vesicles and /or vacuoles were revealed in the gastrodermis of *M. clarii*. The small V1 vesicles probably represent endocytic vesicles into which, partially digested food might be absorbed and move into the gastrodermis cytoplasm. V1 vesicles may then fuse with the V2 vacuoles to form V3 vacuoles. The content of V2 vacuoles may be rich in hydrolytic enzymes produced by RER-Golgi apparatus that complete digestion of the ingested food substance. The V3 vacuoles may contain digested material and residual substances from intracellular digestion. Later, the V3 vacuoles with waste material probably fuse together to accumulate the residual material in larger V4 vacuoles, often found close to the luminal surface and absent from deeper cytoplasmic regions of the gastrodermis. The V4 vacuoles potentially release their contents into the intestinal lumen through exocytosis. The presence of regions containing numerous V4 vacuoles and lacking V2 or V3 vacuoles may indicate that some gastrodermis regions are specialized for digestion and absorption while others are specialized in expelling waste products. Tubular structures were reported in the gastrodermis of *M. congolensis* and *C. kröyeri* (see Arafa et al. 2013; Halton and Stranock 1976 respectively) and were suggested to act as a storage space in which food material can accumulate prior to transfer to the digestive vacuoles supporting the endocytotic mechanism. Such tubular structures were not observed in the gastrodermis of *M. clarii* which means that another mechanism is involved.

A unique feature of the gastrodermis of *M. clarii,* differentiating it from *M. congolensis* and other previously studied monogeneans, is the presence of rounded or oval-shaped vacuoles containing bundles of presumed fibrotic structures. The origin of these structures is unknown and no evidence was found of their occurrence in the intestinal lumen. They may represent degenerated portions of the gastrodermis cytoplasm formed during a regeneration process or a parasite response to toxins, pathogens, autoimmune reactions, and/ or mechanical stress. The accumulation of fibrotic bundles in vacuoles near the luminal surface of the gastrodermis strongly suggest that they can be exocytosed into the intestinal lumen.

No haematin nor melanin granules were observed in the intestinal lumen or gastrodermis of *M. clarii*. Melanin granules were, however, reported in the gut of *M. polypteri* (see Cable et al. 1997) and *M. congolensis* (see Arafa et al. 2013) with similar pigment melanin granules identified in the host’s skin epidermis of these epidermal browsers. Absence of melanin from the intestinal lumen or gastrodermis of *M. clarii* confirms the microhabitat specificity of the two congeneric species: *M. clarii* on the gills and *M. congolensis* on the skin.

## Data Availability

No datasets were generated or analysed during the current study.

## References

[CR1] Allen KM, Tinsley RC (1989) The diet and gastrodermal ultrastructure of polystomatid monogeneans infecting chelonians. Parasitology 2:265–273. 10.1017/S0031182000062181 10.1017/s0031182000062181 2762037

[CR2] Arafa SZ (2011) Ultrastructure of musculature of the marginal hooklets of *Macrogyrodactylus congolensis,* a monogenean skin parasite from the catfish *Clarias gariepinus*. Acta Parasitol 56:122–130. 10.2478/s11686-011-0020-3

[CR3] Arafa SZ, El-Naggar MM, El-Abbassy SA (2009) Mode of attachment and histopathological effects of *Macrogyrodactylus clarii*, a monogenean gill parasite of the catfish Clarias gariepinus, with a report on host response. Acta Parasitol 54:103–112. 10.2478/s11686-009-0026-2

[CR4] Arafa SZ, El-Naggar MM, Kearn GC (2003) Scanning electron microscope observations on the monogenean skin parasite *Macrogyrodactylus congolensis* (Prudhoe, 1957) Yamaguti, 1963. Acta Parasitol 48:163–171

[CR5] Arafa SZ, El-Naggar MM, Kearn GC (2013) Ultrastructure of the digestive system and experimental study of feeding in the monogenean skin and fin parasite *Macrogyrodactylus congolensis*. Acta Parasitol 58:420–433. 10.2478/s11686-013-0169-z 24338302 10.2478/s11686-013-0169-z

[CR6] Arafa SZ, El-Naggar MM, Kearn GC (2014) On some ultrastructural features of the reproductive system of the monogenean parasite *Macrogyrodactylus congolensis* from *Clarias gariepinus* inhabiting the River Nile in Egypt. Acta Parasitol 59:238–246. 10.2478/s11686-014-0236-0 24827092 10.2478/s11686-014-0236-0

[CR7] Bakke TA, Cable J, Harris PD (2007) The biology of gyrodactylid monogeneans: the “Russian-doll killers.” Adv Parasitol 64:161–376. 10.1016/S0065-308X(06)64003-7 17499102 10.1016/S0065-308X(06)64003-7

[CR8] Bogitsh BJ (1993) A comparative review of the flatworm gut with emphasis on the Rhabdcoela and Neodermata. Trans Amer Microsc Soc 112:1–9. 10.2307/3226777

[CR9] Buchmann K (1998) Histochemical characteristics of *Gyrodactylus derjavini* parasitizing the fins of rainbow trout (*Oncorhynchus mykiss*). Folia Parasitol 45:312–318 9868792

[CR10] Buchmann K, Bresciani J (1998) Parasitic infections in pond-reared rainbow trout *Oncorhynchus mykiss* in Denmark. Dis Aquat Org 28:125–138. 10.1016/j.aquaculture.2008.12.030

[CR11] Buchmann K, Køie M, Prentø P (1987) The nutrition of the gill parasitic monogenean *Pseudodactylogyrus anguillae*. Parasitol Res 73:532537. 10.1007/BF00535329 10.1007/BF00535329 3422977

[CR12] Cable J, El-Naggar MM (2021) Gastrodermis ultrastructure of the different life stages of the polyopisthocotylean monogenean gill parasite *Discocotyle sagittata*. Parasitol Res 120:3181–3193. 10.1007/s00436-021-07286-6 34406468 10.1007/s00436-021-07286-6 PMC8397695

[CR13] Cable J, Harris PD, Tinsley RC (1997) Melanin deposition in the gut of the monogenean *Macrogyrodactylus polypteri* Malmberg 1957. Int J Parasitol 27:1323–1331. 10.1016/S0020-7519(97)00089-1 9421719 10.1016/s0020-7519(97)00089-1

[CR14] Cable J, Tinsley RC, Harris PD (2002) Survival, feeding and embryo development of *Gyrodactylus gasterostei* (Monogenea: Gyrodactylidae). Parasitology 124:53–68. 10.1017/S0031182001008861 11811803 10.1017/s0031182001008861

[CR15] Du Preez LH, Verneau O (2020) Eye to eye: classification of conjunctival sac polystomes (Monogenea: Polystomatidae) revisited with the description of three new genera *Apaloneotrema* n. g., *Aussietrema* n. g. and *Fornixtrema* n. g. Parasitol Res 119:4017–4031. 10.1007/s00436-020-06888-w 33043418 10.1007/s00436-020-06888-w

[CR16] El-Naggar MM, Arafa SZ, El-Abbassy SA, Kearn GC (2001) Chaetotaxy of the monogeneans *Macrogyrodactylus clarii *and *M. congolensis* from the gills and skin of the catfish *Clarias gariepinus* in Egypt, with a note on argentophilic elements in the nervous system. Folia Parasitol 48:201–208. 10.14411/fp.2001.033 10.14411/fp.2001.033 11699655

[CR17] El-Naggar MM, Arafa SZ, El-Abbassy SA, Kearn GC, Cable J (2019) Ultrastructure of the anterior adhesive apparatus of the gill parasite *Macrogyrodactylus clarii* and skin parasite *M. congolensis* (Monogenea; Gyrodactylidae) from the catfish *Clarias gariepinus*. Parasitol Int 71:151–159. 10.1016/j.parint.2019.03.005 30853449 10.1016/j.parint.2019.03.005

[CR18] El-Naggar MM, Arafa SZ, El-Abbassy SA, Kearn GC, Cable J (2020) Light and transmission electron microscopy of the haptoral sclerites of the monogenean gill parasite *Macrogyrodactylus clarii*. Parasitol Res 119:4089–4101. 10.1007/s00436-020-06799-w 32683560 10.1007/s00436-020-06799-w

[CR19] El-Naggar MM, Arafa SZ, El-Abbassy SA, Stewart MT, Halton DW (2007) Neuromusculature of *Macrogyrodactylus congolensis*, a monogenean skin parasite of the Nile catfish *Clarias gariepinus*. Parasitol Res 100:265–279. 10.1007/s00436-006-0235-7 16896654 10.1007/s00436-006-0235-7

[CR20] El-Naggar MM, Arafa SZ, Stewart MT, El-Abbassy SA, Halton DW (2004) Neuromusculature of *Macrogyrodactylus clarii*, a monogenean gill parasite of the Nile catfish *Clarias gariepinus* in Egypt. Parasitol Res 94:163–175. 10.1007/s00436-004-1198-1 15322920 10.1007/s00436-004-1198-1

[CR21] El-Naggar MM, Cable J (2007) Ultrastructural observations on the elusive subtegumental cells of the viviparous gill monogenean, *Macrogyrodactylus clarii*. Parasitol Res 101:9–17. 10.1007/s00436-006-0448-9 17265090 10.1007/s00436-006-0448-9

[CR22] El-Naggar MM, Cable J, Arafa SZ, El-Abbassy SA, Kearn GC (2016) Scanning and transmission electron microscopy of the histopathological impact of *Macrogyrodactylus clarii *(Monogenea: Gyrodactylidae) on the gills of catfish, *Clarias gariepinus*. Folia Parasitol 63:017. 10.14411/fp.2016.017 10.14411/fp.2016.017 27311695

[CR23] El-Naggar MM, El-Abbassy SA (2003) Anatomical observations on the viviparous monogenean *Gyrodactylus rysavyi* Ergens, 1973 from the Nile catfish *Clarias gariepinus* in Egypt. Egypt J Zool 40:225–249

[CR24] El-Naggar MM, El-Naggar AM, El-Abbassy SA (2001b) Microhabitat and movement of the viviparous monogeneans *Gyrodactylus alberti,**Macrogyrodactylus clarii* and *M. congolensis* from the Nile catfish *Clarias gariepinus*. J Egypt Ger Soc Zool 35(D):169–187

[CR25] El-Naggar MM, Kearn GC, Hagras AE, Arafa SZ (1999) On some anatomical features of *Macrogyrodactylus congolensis*, a viviparous monogenean ectoparasite of the catfish *Clarias gariepinus* from Nile water. J Egypt Ger Soc Zool 2(D):1–24

[CR26] El-Naggar MM, Khidre AA (1985) Redescription of the monogenean gill parasite *Cichlidogyrus halli typicus* (Price and Kirk, 1967) Paperna, 1979 from *Tilapia* spp. In Egypt. Proceeding from the First International Conference of Applied Sciences. Zagazig University 4:138–159

[CR27] El-Naggar MM, Serag HM (1987) Redescription of *Macrogyrodactylus clarii* Gussev 1961, a monogenean gill parasite of *Clarias lazera* in Egypt. Arab Gulf J Sci Res Agric Biol Sci 5:257–271

[CR28] Fournier A (1978) *Euzetrema knoepffleri*: evidence for a synchronous cycle of gastrodermal activity and an apocrine-like release of the residues of digestion. Parasitology 77:19–26. 10.1017/S0031182000048678

[CR29] Grano-Maldonado MI (2018) Ultrastructure study of the stored lipid reserves in *Gyrodactylus gasterostei *(Monogenea) using confocal and transmission electron microscopy. J Microsc Ultrastruct 6:65–71. 10.4103/JMAU.JMAU_20_18 30221130 10.4103/JMAU.JMAU_20_18 PMC6130249

[CR30] Halton DW, Stranock SD (1976) The fine structure and histochemistry of the caecal epithelium of *Calicotyle kröyeri* (Monogenea: Monopisthocotylea). Int J Parasitol 6:253–263. 10.1016/0020-7519(76)90043-6 1279079 10.1016/0020-7519(76)90043-6

[CR31] Johnsen B, Møkkelgjerd P, Jensen A (2000) The parasite *Gyrodactylus salaris* on salmon parr in Norwegian rivers, status report at the beginning of year. NINA Oppdreagsmeld 617:1–129

[CR32] Kearn GC (1963) Feeding in some monogenean skin parasites: *Entobdella soleae* on *Solea solea* and *Acanthocotyle* sp. on *Raia clavata*. J Mar Biol Ass UK 43:749–766. 10.1017/S0025315400025662

[CR33] Kearn GC, Al-Sehaibani MA, Whittington ID, Evans-Gowing R, Cribb BW (1996) Swallowing of sea water and its role in egestion in the monogenean *Entobdella soleae*, a skin parasite of the common sole (*Solea solea*), with observations on other monogeneans and on a freshwater temnocephalan. J Nat Hist 30:637–646. 10.1080/00222939600770351

[CR34] Kritsky DC, Bourguet D, Spall RD (1994) Fine structure of the gastrodermis of two species of *Gyrodactylus* (Monogenoidea: Polyonchoinea, Gyrodactylidae). Trans Am Microsc Soc 113:43–51. 10.2307/3226578

[CR35] Maduenyane M, Dos Santos QM, Avenant-Oldewage A (2022) First isolation and scanning electron microscopy of haptoral sclerites of *Macrogyrodactylus* (Monogenea). J Helminthol 96(e17):1–9. 10.1017/S0022149X22000037 10.1017/S0022149X22000037 35236526

[CR36] Maduenyane M, Dos Santos QM, Avenant-Oldewage A (2022) Light and scanning electron microscopy of the effects of *Macrogyrodactylus congolensis* (Prudhoe, 1957) on the skin of the African sharptooth catfish *Clarias gariepinus* (Burchell, 1822). J Fish Dis 45:595–602. 10.1111/jfd.13584 35103987 10.1111/jfd.13584

[CR37] Mahdy OA, Sherif AH, Sabry, NM, Attia MM, Abdelsalam M, Prince A, Seida AA (2022) *Macrogyrodactylus* spp. and bacterial co-infection in the farmed African catfish *Clarias gariepinus*. Egypt J Aquat Biol Fish 26:229–242. 10.21608/EJABF.2022.216919

[CR38] Malmberg G (1957) On a new genus of viviparous trematodes. Arkiv För Zoologi 10:17–330

[CR39] Mo TA (1994) Status of *Gyrodactylus salaris* problems and research in Norway. In: Pike AW, Lewis JW (eds) Parasitic Diseases of Fishes. Samara Publishing Limited, UK

[CR40] Paladini G, Cable J, Fioravanti ML, Faria PJ, Cave DD, Shinn AP (2009) *Gyrodactylus orecchiae* sp. n. (Monogenea: Gyrodactylidae) from farmed populations of gilthead seabream (*Sparus aurata*) in the Adriatic Sea. Folia Parasitol 56:21–8. 10.14411/fp.2009.004 10.14411/fp.2009.004 19391328

[CR41] Poddubnaya LG, Hemmingsen W, Reed C, Gibson DI (2015) Ultrastructural characteristics of the caeca of basal polyopisthocotylean monogeneans of the families Chimaericolidae and Hexabothriidae parasitic on cartilaginous fishes. Parasitol Res 114:2599–2610. 10.1007/s00436-015-4464-5 25869960 10.1007/s00436-015-4464-5

[CR42] Truter M, Acosta AA, Weyl OLE, Smit NJ (2021) Novel distribution records and molecular data for species of *Macrogyrodactylus* Malmberg, 1957 (Monogenea: Gyrodactylidae) from *Clarias gariepinus* (Burchell) (Siluriformes: Clariidae) in southern Africa. Folia Parasitol 68:027. 10.14411/fp.2021.027 10.14411/fp.2021.027 34975015

